# Incident Comorbid Diabetes and Outcomes in Multiple Sclerosis: An English Population‐Based Cohort Study

**DOI:** 10.1002/acn3.70548

**Published:** 2026-10-20

**Authors:** Yolanda Lau, Sedigheh Zabihi, Morghan Hartmann, Georgina Mathlin, Sube Banerjee, Ebaa Marouf, Clare Hadley, Claudia Cooper, Ruth Dobson

**Affiliations:** ^1^ Wolfson Institute of Population Health Queen Mary University of London London UK; ^2^ Faculty of Population Health Sciences, Institute of Health Informatics UCL London UK; ^3^ Faculty of Medicine and Health Sciences University of Nottingham Nottingham UK; ^4^ DeNPRU‐QM Patient and Public Engagement Group London UK

**Keywords:** comorbidity, diabetes, health inequalities, multiple sclerosis

## Abstract

**Objective:**

To compare the incidence of diabetes mellitus and its associations with hospitalisation and mortality in adults with multiple sclerosis (MS) and matched controls.

**Methods:**

Using English primary care data from the Clinical Practice Research Datalink, linked to hospital and mortality records, we matched adults with MS with up to 10 controls without MS by age, sex and practice. We excluded individuals with pre‐existing diabetes mellitus. Outcomes included all‐cause hospitalisation (number and duration) and mortality. We used Poisson, negative binomial, linear and Cox proportional hazards models, adjusting for demographic and socioeconomic factors, with interaction terms for ethnicity, deprivation and urbanity.

**Results:**

We included 47,247 individuals with MS and 458,951 matched controls. Over a mean follow‐up of 15.2 years, people with MS had a higher incidence of diabetes mellitus (adjusted incidence rate ratio = 1.16, 95% confidence interval 1.11–1.21). Among people with MS, incident diabetes mellitus was associated with higher hospitalisation rates (adjusted incidence rate ratio = 1.52, 1.42–1.64), longer hospitalisation duration (median 12 vs. 2 days) and increased mortality (adjusted hazard ratio = 1.41, 1.31–1.49). Similar patterns were observed among controls. The association between diabetes mellitus and increased hospitalisation was stronger in rural areas among those with MS and in White groups among controls.

**Interpretation:**

MS was associated with a higher incidence of diabetes mellitus, which was in turn associated with greater hospitalisation and mortality. These findings may support consideration of equitable screening, prevention and management of diabetes mellitus in people living with MS.

## 1 Introduction

Multiple sclerosis (MS) is a chronic neurological disease with increasing prevalence [1]. Almost 3 million people live with MS globally [2], of whom 150,000 live in the United Kingdom [3]. MS is commonly diagnosed in young adulthood. As life expectancy is only marginally reduced in people living with MS [4], comorbid physical and mental health conditions may occur. Comorbidities in MS are associated with worse clinical outcomes, including greater and more rapid disability progression [5, 6], reduced access to disease‐modifying treatments [5], delayed diagnosis and treatment of the comorbid conditions themselves [7] and lower quality of life [8].

Diabetes mellitus (DM) is a common cardiometabolic condition, and a major contributor to morbidity and mortality worldwide [9]. A systematic review estimated Type 2 diabetes prevalence at approximately 5% in people with MS [10]. Existing evidence on comorbid DM and outcomes in people with MS is limited, conflicting and predates widespread use of disease‐modifying therapies. A 2024 review identified three cohort studies examining the association between DM and mortality in people with MS [5], with two reporting increased mortality [11, 12] and one reporting no association [13]. A Danish nationwide cohort study found no difference in the incidence of DM between those with MS and controls over eight years [14]. No studies have examined the influence of comorbid incident DM and MS on hospitalisation, an important contributor to decreased quality of life. Focusing on incident disease captures the impact of potential delayed diagnosis and treatment of DM in MS, which may be diluted across prevalent cases. The potential influence of social determinants of health on these outcomes has not been established.

We addressed this evidence gap using English national linked primary and secondary care data. Our objectives were to: (1) compare incidence of DM between people with MS and controls; (2) examine associations between incident DM and all‐cause hospitalisation (number and duration); (3) examine associations between incident DM and all‐cause mortality and (4) explore whether health inequalities modify these relationships.

## 2 Methods

### 2.1 Study Design and Ethical Approvals

Individuals with MS were matched to controls without MS by sex, year of birth and practice. This project was approved by the Independent Scientific Advisory Committee for MHRA Database Research (protocol number 24_004057). This study was reported in accordance with the Strengthening the Reporting of Observational Studies in Epidemiology (STROBE) guidelines [15] (see Appendix Table 1 in the Supporting Information).

### 2.2 Data Source

Participants were identified from the Clinical Practice Research Datalink (CPRD) Gold and Aurum datasets containing anonymised longitudinal primary care data for approximately 60 million patients (~20% of the UK population) from over 2400 UK general practices.

We linked CPRD data to Hospital Episode Statistics (HES) (Admitted Patient Care [APC], Outpatient, Accident and Emergency), and Office for National Statistics (ONS) death registrations. Area‐level characteristics were derived from the 2011 Rural–Urban Classification at Lower Layer Super Output Area (LSOA) level and patient‐level Index of Multiple Deprivation (IMD).

### 2.3 Exposure and Cohort Definition

The study population was drawn from patients eligible for data linkage across all datasets registered with a primary care practice in England between 2000 and 2023. Exclusion criteria are shown in Table 1.

**TABLE 1:** Exclusion criteria.

Year of birth and registration date	Absence of a record for a year of birth. An age of greater than 115 at the end of follow‐up. An empty or invalid first registration date. A first registration date prior to birth year. A transfer out date prior to their first registration date.
MS patients	Without MS diagnosis date. Without at least 2 years of follow‐up data (defined by patient remained registered with their practice ≥ 2 years after their MS diagnosis) after the first recorded diagnostic code of MS. A first diagnostic MS code recorded within 6 months of registration. If an MS patient was excluded, their matched controls were also excluded.
Covariates	Missing data on sociodemographic variables (age, sex, Index of Multiple Deprivation [IMD] and rural–urban).
Hospitalisation data	Recorded health care episodes in years prior to birth year. Recorded health care episodes with empty or invalid event dates.

#### 2.3.1 Exposure

Diagnosis data for MS and DM were identified using multiple data sources, including CPRD and linked secondary data from HES. Code lists for MS and DM were developed using verified clinical code lists [16, 17] and in consultation with clinicians (Appendix Tables 2–5 in the Supporting Information).

##### 2.3.1.1 MS Cases

The exposed group included adults aged 18–115 years at the time of an MS code recorded in primary care. MS was defined by the presence of two or more MS diagnostic or management codes, or a single MS code accompanied by an appropriate prescription. Codelists for prescriptions were developed using British National Formulary chapters. The date of MS diagnosis (index date) was defined as the first recorded MS code within CPRD.

Matched controls: Each individual with MS was matched to up to 10 controls without MS, exactly on all three variables: general practice, sex and year of birth, without replacement, selected at random using a fixed seed. Controls were assigned the same index date as their matched MS case. Matching was performed before study‐specific exclusion criteria were applied, in line with previous published CPRD analyses, because, as standard practice, electronic health record data providers, including CPRD, do not permit access to the entire dataset.

##### 2.3.1.2 DM

DM was defined by ≥ 1 diagnostic or management code for Type 1, Type 2 or unspecified DM, with or without an antidiabetic prescription. DM type was identified from type‐specific codes. Those with only gestational diabetes were excluded. DM was classified as suspected (single code, or prescription without diagnosis) or confirmed (≥ 2 diagnoses, or a diagnosis with prescription). The diagnosis date was the first recorded code or prescription. Individuals with DM (suspected or confirmed) recorded prior to the index date (i.e., prevalent DM) were excluded.

#### 2.3.2 Outcomes

A 6‐month wash‐out period after MS diagnosis was applied to ensure that outcomes were not related to the diagnostic process.

- All‐cause hospitalisation episodes and duration: Any hospital admission and the duration of all hospital admissions during the follow‐up period were calculated, using HES APC.
- All‐cause mortality: Date of death was obtained from ONS death registration records.

#### 2.3.3 Covariates

Covariates included age, sex, ethnicity (defined using self‐reported UK Census categories), IMD (a measure of socioeconomic status), urban/rural classification (the practice the patient was registered to) and smoking status. The IMD is an area‐level measure of relative socioeconomic deprivation in England, derived at LSOA level, combining seven weighted domains: income, employment, education, health, crime, barriers to housing and services and living environment [18]. Baseline hypertension and BMI were compared between groups, and any that differed were included as a covariate in a sensitivity analysis.

### 2.4 Missing Data

Those with missing age, sex, IMD, or rural/urban classification were excluded; missing ethnicity data were retained as ‘unknown.’ Differences between those with and without missing data were examined using chi‐squared tests to assess whether missingness was plausibly consistent with a missing‐at‐random (MAR) assumption. Significant differences were observed between those with and without smoking status, so smoking status was imputed using multiple imputation by chained equations (five imputed datasets).

### 2.5 Statistical Analysis

The analysis plan was pre‐registered [19]. Chi‐squared tests and *t*‐tests were used to compare baseline characteristics between individuals with MS and their matched controls. Given their role as potential confounders, baseline hypertension (any hypertension diagnostic code before the index date) and BMI (most recent valid measurement before the index date) were also compared. Models were adjusted for age, sex, ethnicity, IMD, rural/urbanity, smoking status and random effects for practices.

#### 2.5.1 Objective 1: Incidence of DM

We calculated incidence rates of DM by patient‐years. Multivariable Poisson regression models were used to compare the incidence of suspected or confirmed DM diagnosis between groups. Results were reported as incidence rate ratio (IRR). As a sensitivity analysis, we also fitted a cause‐specific Cox proportional hazards model, censoring at death or study exit.

#### 2.5.2 Objective 2: All‐Cause Hospitalisation Episodes and Duration

We used negative binomial and linear regression models to examine differences in hospitalisation episodes (number and duration respectively) between groups. Separate models were conducted for MS and controls. Results were reported as IRR and coefficients.

#### 2.5.3 Objective 3: All‐Cause Mortality

Adjusted Cox models compared time to all‐cause mortality between individuals with and without DM, with incident DM also modelled as a time‐varying exposure and the proportional hazards assumption assessed using Schoenfeld residuals plots and test. Analyses were conducted separately for MS and controls, and results were reported as hazard ratio (HR).

To examine whether associations differed between MS and controls, interaction terms between MS and DM were fitted across objectives.

#### 2.5.4 Objective 4: Potential Health Inequalities

Interaction terms were added to models to examine if ethnicity, IMD or urbanity modified associations with hospitalisation and mortality. We fitted and tested interactions using the likelihood ratio test. Results were stratified by the factor of interaction (e.g., rural or urban area) where the interaction term was statistically significant.

### 2.6 Subgroup and Sensitivity Analyses

Subgroup analysis was conducted to examine Type 1 and Type 2 DM. To assess whether the association between MS and incident DM differed by DM type, an interaction term between MS status and DM type was fitted.

Four sensitivity analyses were conducted. First, we restricted the MS group to those with MS diagnosed from 2002 onward, reflecting the implementation of McDonald diagnostic criteria and increasing disease‐modifying therapy use in clinical practice. Second, we defined incident DM based on confirmed DM only, while individuals with suspected DM were reclassified as no DM. Finally, we conducted a post hoc sensitivity analysis adjusting the primary models for baseline hypertension.

## 3 Results

### 3.1 Study Characteristics

After applying inclusion and exclusion criteria, the final dataset comprised 47,247 participants with MS and 458,951 matched controls (Figure 1). The mean age was 43.8 (SD 13.6), and 29.6% (*n* = 149,636) were male (Table 2). Compared with controls, individuals with MS were of similar age (mean 43.9 [SD 13.6] vs. 43.8 years [SD 13.6]) but had shorter follow‐up (mean 14.3 [SD 6.4] vs. 15.3 years [SD 6.7]). They were more likely to be from a White ethnic group (93.0% vs. 84.5%) and to be current or ex‐smokers. Diabetes (confirmed or suspected) was of similar prevalence in the two groups (MS 4.5% vs. controls 4.7%). At baseline, 16.1% of people with MS and 12.9% of controls had hypertension (*p* = 0.02). BMI did not differ between groups (mean 25.5 [SD 5.3] in MS vs. 25.4 [SD 5.3] in controls; *p* = 0.28).

**FIGURE 1:**
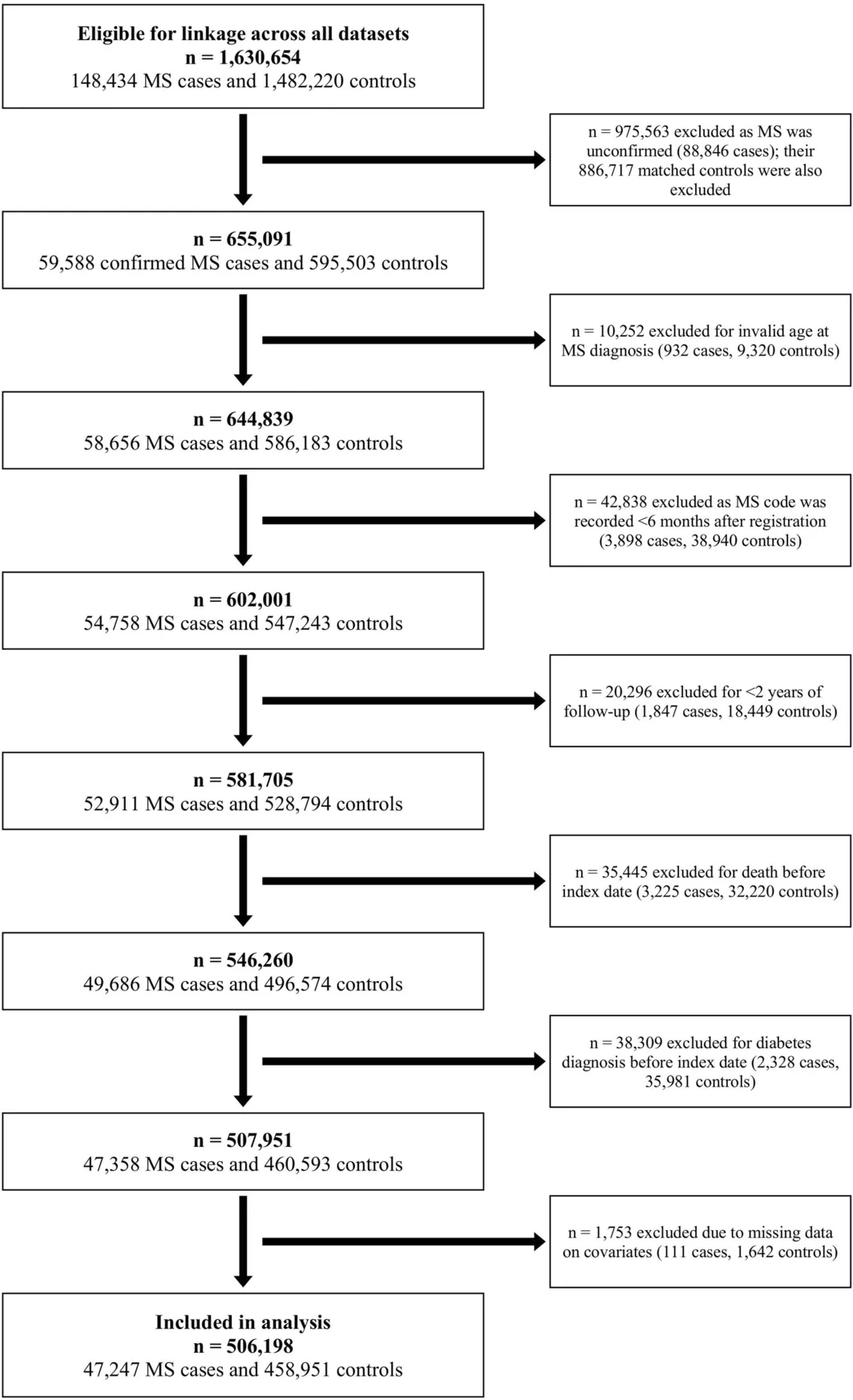
Study flowchart.

**TABLE 2:** Characteristics of the population at the time of entering the study (index date).

	Total (*n* = 506,198)	MS (47,247)	Controls (458,951)	*p*
Age	43.8 (13.6)	43.9 (13.6)	43.8 (13.6)	0.008*
Sex (male)	149,636 (29.6%)	14,014 (29.7%)	135,622 (29.6%)	0.62
Ethnicity				
White	431,839 (85.3%)	43,917 (93.0%)	387,922 (84.5%)	< 0.001*
Black	14,361 (2.8%)	897 (1.9%)	13,464 (2.9%)	
South Asian	13,449 (2.7%)	867 (1.8%)	12,582 (2.7%)	
Asian	10,462 (2.1%)	405 (0.9%)	10,057 (2.2%)	
Mixed/multiple	5230 (1.0%)	475 (1.0%)	4755 (1.0%)	
Other	2377 (0.5%)	65 (0.1%)	2312 (0.5%)	
Unknown	28,480 (5.6%)	621 (1.3%)	27,859 (6.1%)	
Smoking status				
Current smoker	70,746 (14.0%)	8546 (18.1%)	62,200 (13.6%)	< 0.001*
Ex‐smoker	54,021 (10.7%)	6759 (14.3%)	47,262 (10.3%)	
Non‐smoker	165,517 (32.7%)	15,627 (33.1%)	149,890 (32.7%)	
Missing data	215,914 (42.7%)	16,315 (34.5%)	199,599 (43.5%)	
IMD				
Quintile 1^a^	115,590 (22.8%)	10,627 (22.5%)	104,963 (22.9%)	0.002*
Quintile 2	113,258 (22.4%)	10,736 (22.7%)	102,522 (22.3%)	
Quintile 3	104,610 (20.7%)	9816 (20.8%)	94,794 (20.7%)	
Quintile 4	94,694 (18.7%)	9009 (19.1%)	85,685 (18.7%)	
Quintile 5	78,046 (15.4%)	7059 (14.9%)	70,987 (15.5%)	
Rural–urban classification				
Urban	430,206 (85.0%)	40,162 (85.0%)	390,044 (85.0%)	0.92
Rural	75,992 (15.0%)	7085 (15.0%)	68,907 (15.0%)	
	430,206 (85.0%)	40,162 (85.0%)	390,044 (85.0%)	
Follow‐up duration	15.2 (6.7)	14.3 (6.4)	15.3 (6.7)	< 0.001*
Diagnosis of DM				
No	482,359 (95.3%)	45,128 (95.5%)	437,231 (95.3%)	< 0.001*
Confirmed	13,432 (2.7%)	1074 (2.3%)	12,358 (2.7%)	
Suspected	10,407 (2.1%)	1045 (2.2%)	9362 (2.0%)	

^a^ Five quintiles, with Quintile 1 representing the most socioeconomic deprivation and Quintile 5 least socioeconomic deprivation.

* *p* < 0.05.

Missing data on smoking status were associated with age, ethnicity, IMD and MS status (all *p* < 0.001), suggesting data were not missing completely at random.

### 3.2 Objective 1: Incidence of DM

Over a mean follow‐up of 15.2 years, 2119 (4.48%) participants with MS and 21,720 (4.73%) of controls developed DM (suspected or confirmed DM). The crude incidence rate of DM was 3.14 per 1000 person‐years among participants with MS, and 3.00 per 1000 person‐years among controls.

People with MS had a higher incidence of DM compared with controls (unadjusted IRR = 1.08, 95% confidence interval [CI]: 1.03–1.13, *p* < 0.001; adjusted IRR [aIRR] = 1.16, 95% CI: 1.11–1.21, *p* < 0.001). Results were consistent in sensitivity analyses (Appendix Table 6 in the Supporting Information). Results were unchanged after further adjustment for baseline hypertension (aIRR = 1.15, 95% CI: 1.04–1.21, *p* < 0.001). The cause‐specific Cox proportional hazards model found a similar estimate (aHR = 1.15, 95% CI: 1.11–1.21, *p* < 0.001).

Among 23,839 participants with incident DM, 8533 (35.79%) had information on the type of DM (Type 1: *n* = 585, Type 2: *n* = 7948). In subgroup analyses by DM known type, this association remained significant in both types, with slightly higher IRR in Type 1 DM (Appendix Table 7 in the Supporting Information).

### 3.3 Objective 2: All‐Cause Hospitalisation Episodes and Duration

#### 3.3.1 Hospitalisation Episodes

Among people with MS, those with incident DM had more hospitalisation episodes in unadjusted (IRR = 2.83, 95% CI: 2.44–3.28, *p* < 0.001) and adjusted models (aIRR = 1.52, 95% CI: 1.42–1.64, *p* < 0.001). Results remained significant in sensitivity analyses restricting to MS diagnosed from 2002 onward and when incident DM was restricted to confirmed DM (Appendix Table 8 in the Supporting Information). Results were unchanged after further adjustment for hypertension (aIRR = 1.53, 95% CI: 1.42–1.64, *p* < 0.001).

Among controls, those with incident DM had more hospitalisation episodes in unadjusted (IRR = 5.17, 95% CI 4.90–5.46, *p* < 0.001) and adjusted models (aIRR = 2.36, 95% CI 2.29–2.44, *p* < 0.001; further adjustment for hypertension aIRR 2.35, 2.27–2.45, *p* < 0.001).

The association between incident DM and hospitalisation episodes was stronger in controls than the MS group (aIRR = 0.43, 95% CI 0.40–0.47; *p* < 0.001).

In both MS group and controls, subgroup analyses found that Type 1 and Type 2 DM were associated with more hospitalisation episodes (Appendix Table 9 in the Supporting Information) but the difference between groups was not significant (controls: *β* = −0.11, 95% CI −0.37 to 0.05; *p* = 0.15; MS: *β* = −0.09, 95% CI −0.16 to 0.35; *p* = 0.37).

#### 3.3.2 Hospitalisation Duration

Among people with MS, median duration was 12 days (interquartile range [IQR] 0–83) for those with incident DM and 2 days (IQR 0–22) for those without incident DM. This was a significant difference between people with MS with and without incident DM in both unadjusted (β = 0.93, 95% CI: 0.89–0.97, *p* < 0.001) and adjusted models (*β* = 0.89, 95% CI: 0.78–1.01, *p* < 0.001). Results remained significant in sensitivity analyses on MS diagnosed from 2002 onward and when incident DM was restricted to confirmed DM (Appendix Table 10 in the Supporting Information), and when further adjusted for hypertension (*β* = 0.92, 95% CI: 0.89–0.94, *p* < 0.001).

Among controls, median duration was 10 days (IQR 2–30) for those with incident DM and 1 day (IQR 0–5) for those without incident DM; this was a significant difference in both unadjusted (*β* = 1.08, 95% CI: 1.03–1.12, *p* < 0.001) and adjusted models (*β* = 0.62, 95% CI: 0.61–0.63, *p* < 0.001; model further adjusting for hypertension; *β* = 0.62, 95% CI 0.60–0.63, *p* < 0.001).

In a model combining both cohorts with MS × incident DM interaction term, the association between incident DM and hospitalisation duration was stronger among controls than among people with MS (adjusted *β*: −0.21, 95% CI −0.35 to −0.07, *p* < 0.001).

In both MS group and controls, subgroup analyses found both Type 1 and Type 2 DM were associated with longer hospitalisation duration (Appendix Table 11 in the Supporting Information), with no significant difference between Type 1 and Type 2 DM (controls *β* = −0.13, 95% CI −0.38 to 0.09, *p* = 0.21; MS: *β* = −0.08, 95% CI −0.68 to 0.37, *p* = 0.73).

### 3.4 Objective 3: All‐Cause Mortality

Of the 47,247 participants with MS, 11,388 (24.11%) died during follow‐up. Of the 458,951 controls, 47,098 (10.26%) died.

Schoenfeld residual tests indicated non‐proportional hazards for age (*p* < 0.001), ethnicity (*p* = 0.02) and the overall model (*p* < 0.001) (Appendix Figure 1 in the Supporting Information).

Among people with MS, incident DM was associated with increased all‐cause mortality (adjusted HR = 1.08, 95% CI: 1.01–1.15, *p* = 0.02). When modelled as a time‐varying exposure, incident DM was associated with 41% higher hazard of mortality (HR = 1.41, 95% CI: 1.31–1.49, *p* < 0.001). It remained significant when restricted to MS diagnosed from 2002 onwards (adjusted hazard ratio [aHR] = 1.09, 95% CI 1.03–1.18, *p* = 0.01), when restricting to confirmed incident DM (aHR = 1.69, 95% CI 1.53–1.88, *p* < 0.001), and when further adjusting for hypertension (aHR = 1.68, 95% CI: 1.51–1.87, *p* < 0.001). Subgroup analyses by DM type showed significant associations with mortality in MS (Type 2 only) and controls (Appendix Table 12 in the Supporting Information).

Among controls, incident DM was associated with mortality relative to those without (HR = 1.25, 95% CI: 1.21–1.34, *p* < 0.001). When modelled as a time‐varying exposure, incident DM was associated with a 69% higher hazard of mortality (HR = 1.69, 95% CI: 1.51–1.88, *p* < 0.001). It remained significant when further adjusting for hypertension (aHR = 1.68, 95% CI: 1.51–1.90, *p* < 0.001).

The association between incident DM and mortality did not differ between individuals with MS and controls, either in the main model (HR = 1.11, 95% CI 0.73–1.41, *p* = 0.44) or when DM was modelled as a time‐varying exposure (HR = 1.01, 95% CI 0.88–1.39, *p* = 0.63).

### 3.5 Objective 4: Findings From Interaction Analyses

Table 3 shows the results of interaction analyses, and Appendix Tables 13–17 in the Supporting Information show results of stratified analyses. Interaction analyses on the adjusted models found no evidence that ethnicity, IMD, or urbanity modified the relationship between incident DM and group (MS or control). In controls, the association between incident DM and hospitalisation episodes and duration was stronger in White than non‐White groups, while among people with MS, this association was stronger (for number of episodes only) among those living in rural areas. Among controls from least deprived groups and those in rural areas, there was a stronger association between incident DM and mortality.

**TABLE 3:** Results of interaction analyses relating to objectives comparing MS and control groups (*p* values).

	Ethnicity	IMD	Urban/rural
Objective 1: Incidence of DM	*p* = 0.78	*p* = 0.17	*p* = 0.66
Objective 2: Hospitalisation episodes	MS: *p* = 0.15 Controls: *p* = 0.001*	MS: *p* = 0.19 Controls: *p* = 0.78	MS: *p* = 0.02* Controls = 0.71
Objective 2: Hospitalisation duration	MS: *p* = 0.34 Controls: *p* < 0.001*	MS: *p* = 0.75 Controls = 0.36	MS: *p* = 0.19 Controls: *p* = 0.20
Objective 3: Mortality	MS: *p* = 0.38 Controls: *p* = 0.14	MS: *p* = 0.14 Controls: *p* = 0.03*	MS: *p* = 0.58 Controls: *p* = 0.04*

* *p* < 0.05.

## 4 Discussion

We found that people with MS had a higher incidence of DM than matched controls. Among people with MS, incident DM was associated with more and longer hospitalisations and mortality compared with those who did not develop DM. Both people with MS and controls with DM had higher mortality, with no significant difference between these groups. The relationship between DM and greater hospitalisation was stronger in rural areas among those with MS; in controls it was stronger in the White ethnic group, and the relationship of DM with mortality was stronger in the least deprived groups and those in rural areas.

Our finding that MS was associated with a higher incidence of DM echoes earlier research showing this group are also at increased risk of cardiovascular disease [20, 21]. In both individuals with MS and controls, DM was associated with longer hospitalisations, aligning with a study of prevalent DM [22]. A large Canadian study also linked prevalent DM to increased mortality in both people with MS (aHR = 1.47) and matched controls [11]; and two cohort studies reported increased mortality risk among people with MS and prevalent DM, with aHRs of approximately 1.39–1.48 [5].

The mechanisms underlying this association are not fully understood [23], but there are several possibilities. MS and diabetes share risk factors, including physical inactivity [24], obesity [25] and immune system dysregulation [26]. For Type 1 diabetes, shared autoimmune pathways and genetic susceptibility with MS may explain their co‐occurrence [27]. For Type 2 diabetes, the association more likely reflects metabolic and lifestyle factors: reduced physical activity related to MS disability can promote insulin resistance, which is more common in people with MS than in controls [28] and is a precursor to Type 2 diabetes.

There were stronger associations between DM and hospitalisation episodes and duration in controls than in people with MS. Perhaps MS‐related hospitalisations [29, 30] attenuate the relationship between incident diabetes and hospitalisation. Closer monitoring among people with MS may lead to better management of DM‐related complications, although this is not reflected in the higher mortality rates.

We found that health inequalities may modify associations between comorbid DM and outcomes. Among individuals with MS, the association between DM and hospitalisation episodes was stronger in those living in rural areas. However, despite evidence that lower socioeconomic status is associated with faster disability progression [31] and ethnicity with greater symptom burden and overall disability [32], neither IMD nor ethnicity modified these associations. Perhaps the frequent health care contacts among people with MS attenuate inequalities in accessing preventive care, related to deprivation and ethnicity.

### 4.1 Strengths and Limitations

We used a large, population‐based matched cohort from linked primary and secondary care data, enabling robust estimations of outcomes. Matching on age, sex and practice reduced confounding, and additional adjustment for socio‐demographic factors and smoking strengthened internal validity. Sensitivity analyses—including restriction to incident MS after the McDonald criteria and confirmed diagnoses—supported robustness of findings. Modelling DM as a time‐varying exposure reduced immortal time bias.

Limitations should be acknowledged. First, CPRD relies on routinely collected data, so information may be incomplete (particularly ethnicity data), and individuals who are less engaged with health services may be under‐represented. Second, subgroup and stratified analyses were based on relatively small sample sizes and should be interpreted with caution. Third, diabetes type was unrecorded in 64% of individuals with incident DM. Across several UK primary care databases, type‐specific codes have been shown to be subject to miscoding and misclassification [33, 34]. We did not analyse HbA1c as it was not reliably available. Fourth, individuals who develop diabetes may differ systematically from those who do not in baseline health status and comorbidity burden [35]. Although diabetes was modelled as a time‐varying exposure to reduce immortal time bias, residual confounding by unmeasured baseline health and comorbidity cannot be excluded. Fifth, people with MS often have more frequent contact with healthcare services than the general population, which may increase detection of asymptomatic or early diabetes and could partly account for the higher observed incidence of diabetes in the MS group. Similarly, the greater comorbidity burden in people with MS may partly explain their longer observed duration of hospitalisation. However, this does not explain the increased mortality associated with comorbid DM, underlining the importance of adequate management of comorbid DM where detected. The index date reflects the first recorded MS code in CPRD rather than first clinical diagnosis, and postdates the true onset of MS. Unmeasured disease duration may influence both diabetes risk and outcomes. We attempted to mitigate this by excluding individuals whose first MS code was within 6 months of registration. Finally, as MS and diabetes epidemiology varies by geography and ethnicity—including latitudinal gradients in MS and Type 1 diabetes [36]—the incidence estimates from our predominantly White, English cohort may not generalise to other populations.

## 5 Conclusion

Individuals with MS had a higher incidence of DM compared with controls. In people with MS, incident DM was associated with higher rates of all‐cause hospitalisation and mortality, with similar patterns in controls. The association between comorbid DM and outcomes in MS varied by health inequalities. Although future studies are needed, equity‐informed DM screening, prevention and management may benefit people with MS.

## Author Contributions

S.B., G.M., C.C., R.D. and S.Z. contributed to the conception of the study. Y.L., G.M., C.C., S.B., R.D. and S.Z. contributed to the study design. Y.L., S.Z. and M.H. conducted the data analysis and data cleaning. Y.L., C.C., E.M., C.H. and R.D. contributed to the interpretation of the data. Y.L. drafted the initial manuscript. E.M. and C.H. provided patient and public involvement (PPI) input. C.C. and R.D. supervised the study. All authors critically reviewed and revised the manuscript for important intellectual content and approved the final version for submission.

## Funding

This work was supported by the National Institute for Health and Care Research, NIHR206110.

## Conflicts of Interest

The authors declare no conflicts of interest.

## Supporting information


**Appendix Table 1:** STROBE statement checklist.
**Appendix Table 2:** Code lists for multiple sclerosis.
**Appendix Table 3:** Code lists for prescriptions used to support a diagnosis of MS.
**Appendix Table 4:** Code lists for diabetes mellitus.
**Appendix Table 5:** Code lists for prescriptions used to support a diagnosis of diabetes.
**Appendix Table 6:** Objective 1: Sensitivity analyses.
**Appendix Table 7:** Objective 1: Subgroup analyses.
**Appendix Table 8:** Objective 2: Sensitivity analyses, hospitalisation episode.
**Appendix Table 9:** Objective 2: Subgroup analyses on hospitalisation episodes.
**Appendix Table 10:** Objective 2: SENSITIVITY analyses on hospitalisation duration.
**Appendix Table 11:** Objective 2: Subgroup analyses, hospitalisation duration.
**Appendix Table 12:** Objective 3: Subgroup analyses (time‐varying model).
**Appendix Table 13:** Objective 2: Stratified analyses, hospitalisation episodes in controls.
**Appendix Table 14:** Objective 2: Stratified analyses, hospitalisation duration in controls.
**Appendix Table 15:** Objective 2: Stratified analyses, hospitalisation episodes in the MS group.
**Appendix Table 16:** Objective 3: Stratified analyses, mortality in controls.
**Appendix Table 17:** Objective 3: Stratified analyses, mortality in controls.
**Appendix Figure 1:** Schoenfeld residual plot.

## Data Availability

The data used in this study were obtained from the Clinical Practice Research Datalink (CPRD) under licence and cannot be shared by the authors. Data are available from CPRD (https://www.cprd.com) subject to protocol approval and the relevant licence agreements. The study protocol and analysis code are available from the corresponding author on reasonable request.
