# Supplementary material for: Incident Comorbid Diabetes and Outcomes in Multiple Sclerosis: An English Population‐Based Cohort Study
Source: Ann Clin Transl Neurol. 2026 Oct 20:10.1002/acn3.70548. Online ahead of print. doi: 10.1002/acn3.70548 (PMC13682410; doi:10.1002/acn3.70548)
Supplement: Supplementary file 1 [file ACN3-9999-0-s001.docx]

**APPENDIX**

**Table 1. STROBE statement checklist**

|  | Item No. | Recommendation | This study |
| --- | --- | --- | --- |
| **Title and abstract** | 1 | (*a*) Indicate the study’s design with a commonly used term in the title or the abstract | See title |
|  |  | (*b*) Provide in the abstract an informative and balanced summary of what was done and what was found | See abstract |
| Introduction | | | |
| Background/rationale | 2 | Explain the scientific background and rationale for the investigation being reported | Introduction, paragraphs 1–2 |
| Objectives | 3 | State specific objectives, including any prespecified hypotheses | Introduction, final paragraph; pre-registered analysis plan |
| Methods | | | |
| Study design | 4 | Present key elements of study design early in the paper | Methods, ‘Study design and ethical approvals’ |
| Setting | 5 | Describe the setting, locations, and relevant dates, including periods of recruitment, exposure, follow-up, and data collection | Methods, ‘Data source’ |
| Participants | 6 | *Cohort study*—Give the eligibility criteria, and the sources and methods of selection of participants. Describe methods of follow-up | Methods, ‘Exposure and cohort definition’; Table 1 |
|  |  | *Cohort study*—For matched studies, give matching criteria and number of exposed and unexposed | Methods, ‘Matched controls’; Results and Table 2 |
| Variables | 7 | Clearly define all outcomes, exposures, predictors, potential confounders, and effect modifiers. Give diagnostic criteria, if applicable | Methods, ‘Exposure’, ‘Outcomes’, ‘Covariates’ |
| Data sources/ measurement | 8* | For each variable of interest, give sources of data and details of methods of assessment (measurement). Describe comparability of assessment methods if there is more than one group | Methods, ‘Data source’ and ‘Exposure’; codelists in Appendix Tables 2–5 |
| Bias | 9 | Describe any efforts to addresis potential sources of bias | Methods, ‘Outcomes’ (six-month wash-out) and ‘Statistical analysis’ (DM as time-varying exposure) |
| Study size | 10 | Explain how the study size was arrived at | Methods, ‘Data source’, ‘Exposure and cohort definition)’, and Figure 1 |
| Quantitative variables | 11 | Explain how quantitative variables were handled in the analyses. If applicable, describe which groupings were chosen and why | Methods, ‘Covariates’ and ‘Statistical analysis’ |
| Statistical methods | 12 | (*a*) Describe all statistical methods, including those used to control for confounding | Methods, ‘Statistical analysis’ |
|  |  | (*b*) Describe any methods used to examine subgroups and interactions | Methods, ‘Objective 4’ and ‘Subgroup and sensitivity analyses’ |
|  |  | (*c*) Explain how missing data were addressed | Methods, ‘Missing data’ |
|  |  | (*d*) *Cohort study*—If applicable, explain how loss to follow-up was addressed  *Case-control study*—If applicable, explain how matching of cases and controls was addressed  *Cross-sectional study*—If applicable, describe analytical methods taking account of sampling strategy | Not applicable |
|  |  | (*e*) Describe any sensitivity analyses | Methods, ‘Subgroup and sensitivity analyses’ |
| Results |  |  |  |
| Participants | 13* | (a) Report numbers of individuals at each stage of study—eg numbers potentially eligible, examined for eligibility, confirmed eligible, included in the study, completing follow-up, and analysed | Results, first paragraph; Figure 1 |
|  |  | (b) Give reasons for non-participation at each stage | Figure 1; Table 1 |
|  |  | (c) Consider use of a flow diagram | Figure 1 |
| Descriptive data | 14* | (a) Give characteristics of study participants (eg demographic, clinical, social) and information on exposures and potential confounders | Results; Table 2 |
|  |  | (b) Indicate number of participants with missing data for each variable of interest | Results; Table 2 |
|  |  | (c) *Cohort study*—Summarise follow-up time (eg, average and total amount) | Results; Table 2 |
| Outcome data | 15* | *Cohort study*—Report numbers of outcome events or summary measures over time | Results, ‘Objective 1–3’ |
|  |  | *Case-control study—*Report numbers in each exposure category, or summary measures of exposure | Results; adjusted/unadjusted estimates with 95% confidence interval throughout |
|  |  | *Cross-sectional study—*Report numbers of outcome events or summary measures | Table 2 footnote |
| Main results | 16 | (*a*) Give unadjusted estimates and, if applicable, confounder-adjusted estimates and their precision (eg, 95% confidence interval). Make clear which confounders were adjusted for and why they were included | Results (absolute incidence per 1,000 person-years) |
|  |  | (*b*) Report category boundaries when continuous variables were categorized | Results; Appendix Tables 6–16 |
|  |  | (*c*) If relevant, consider translating estimates of relative risk into absolute risk for a meaningful time period | Results, first paragraph; Figure 1 |
| Other analyses | 17 | Report other analyses done—eg analyses of subgroups and interactions, and sensitivity analyses | Figure 1; Table 1 |
| Discussion |  |  |  |
| Key results | 18 | Summarise key results with reference to study objectives | Discussion, first paragraph |
| Limitations | 19 | Discuss limitations of the study, taking into account sources of potential bias or imprecision. Discuss both direction and magnitude of any potential bias | Discussion, ‘Strengths and limitations’ |
| Interpretation | 20 | Give a cautious overall interpretation of results considering objectives, limitations, multiplicity of analyses, results from similar studies, and other relevant evidence | Discussion and Conclusion |
| Generalisability | 21 | Discuss the generalisability (external validity) of the study results | Discussion |
| Other information |  |  |  |
| Funding | 22 | Give the source of funding and the role of the funders for the present study and, if applicable, for the original study on which the present article is based | Acknowledgements |

**Table 2.** Code lists for Multiple Sclerosis

**READ codes**

**Term**

Multiple sclerosis monitoring first letter

Multiple sclerosis monitoring telephone invitation

Benign multiple sclerosis

Generalised multiple sclerosis

Multiple sclerosis of the spinal cord

Management of multiple sclerosis in onset phase

Spec serv for pat with multiple sclerosis - enh serv admin

Multiple sclerosis monitoring administration

Management of multiple sclerosis in early disease phase

Multiple sclerosis review

Multiple sclerosis of the brain stem

Management of multiple sclerosis in stable disability phase

Referral to community multiple sclerosis team

Suspected multiple sclerosis

Relapsing and remitting multiple sclerosis

Multiple sclerosis NOS

Multiple sclerosis care plan agreed

Multiple sclerosis monitoring second letter

Multiple sclerosis

Exacerbation of multiple sclerosis

Secondary progressive multiple sclerosis

Primary progressive multiple sclerosis

Management of multiple sclerosis in palliative phase

Multiple sclerosis review declined

Multiple sclerosis multidisciplinary review

Kurtzke multiple sclerosis rating scale

Disseminated sclerosis

Concentric sclerosis

EDSS - Expanded disability status scale

[X]Demyelinating diseases of the central nervous system

**SNOMED**

**Term**

Clinically isolated syndrome

Multiple sclerosis NOS

Secondary progressive multiple sclerosis

Relapsing remitting multiple sclerosis

Primary progressive multiple sclerosis

Malignant multiple sclerosis

Benign multiple sclerosis

Exacerbation of multiple sclerosis

Generalised multiple sclerosis

Multiple sclerosis of the spinal cord

Multiple sclerosis of the brain stem

Multiple sclerosis

Transverse myelitis

Multiple sclerosis monitoring third letter

Multiple sclerosis monitoring telephone invitation

Multiple sclerosis monitoring second letter

Multiple sclerosis monitoring first letter

Multiple sclerosis monitoring administration

Specialised services for patients with multiple sclerosis enhanced services administration

Multiple sclerosis review declined

Referral to community multiple sclerosis team

Multiple sclerosis care plan agreed

Management of multiple sclerosis in palliative phase

Management of multiple sclerosis in progressive disability phase

Management of multiple sclerosis in stable disability phase

Management of multiple sclerosis in early disease phase

Management of multiple sclerosis in onset phase

Multiple sclerosis multidisciplinary review

Multiple sclerosis review

Suspected multiple sclerosis

[RFC] Multiple sclerosis

[RFC] Multiple sclerosis

Suspected multiple sclerosis

Referral by Multiple Sclerosis nurse specialist

Multiple sclerosis - personal health plan

Multiple sclerosis questionnaire completed

Multiple sclerosis monitoring telephone invite

Multiple sclerosis monitoring third letter

Multiple sclerosis monitoring first letter

Multiple sclerosis monitoring administration

Multiple sclerosis - relapsing remitting

Multiple sclerosis - secondary progressive

Multiple sclerosis - primary progressive

Possible MS

Remittent-progressive multiple sclerosis

Progressive relapsing multiple sclerosis

Multiple sclerosis clinic

Multiple sclerosis (MS) primary progressive

Multiple sclerosis (MS) relapsing remitting

Multiple sclerosis (MS) secondary progressive

**ICD-10**

**Term**

Multiple Sclerosis

**Table 3.** Code lists for prescriptions for considering diagnosis of MS

| Alemtuzumab | Diroximel fumarate |
| --- | --- |
| interferon beta-1a | Fingolimod |
| Cladribine | Glatiramer acetate |
| Dimethyl fumarate | Natalizumab |
| Ocrelizumab | Ofatumumab |
| Teriflunomide | Siponimod |
| Ponesimod | Peginterferon beta 1a |
| Ozanimod |  |

**Table 4.** Code lists for Diabetes Mellitus

| **READ codes** | |
| --- | --- |
| **Code** | **Term** |
| C10D.00 | Diabetes mellitus autosomal dominant type 2 |
| 66An.00 | Diabetes type 1 review |
| 66Ao.00 | Diabetes type 2 review |
| ZC2C900 | Dietary advice for type I diabetes |
| ZC2CA00 | Dietary advice for type II diabetes |
| C10FK00 | Hyperosmolar non-ketotic state in type 2 diabetes mellitus |
| C10FJ00 | Insulin treated Type 2 diabetes mellitus |
| C10E.00 | Type 1 diabetes mellitus |
| C10E800 | Type 1 diabetes mellitus - poor control |
| C10P011 | Type 1 diabetes mellitus in remission |
| C10EH00 | Type 1 diabetes mellitus with arthropathy |
| C10EF00 | Type 1 diabetes mellitus with diabetic cataract |
| C10EP00 | Type 1 diabetes mellitus with exudative maculopathy |
| C10E600 | Type 1 diabetes mellitus with gangrene |
| C10EQ00 | Type 1 diabetes mellitus with gastroparesis |
| C10EE00 | Type 1 diabetes mellitus with hypoglycaemic coma |
| C10EM00 | Type 1 diabetes mellitus with ketoacidosis |
| C10EN00 | Type 1 diabetes mellitus with ketoacidotic coma |
| C10EB00 | Type 1 diabetes mellitus with mononeuropathy |
| C10E300 | Type 1 diabetes mellitus with multiple complications |
| C10ED00 | Type 1 diabetes mellitus with nephropathy |
| C10E200 | Type 1 diabetes mellitus with neurological complications |
| C108J12 | Type 1 diabetes mellitus with neuropathic arthropathy |
| C10E100 | Type 1 diabetes mellitus with ophthalmic complications |
| C10EG00 | Type 1 diabetes mellitus with peripheral angiopathy |
| C10EL00 | Type 1 diabetes mellitus with persistent microalbuminuria |
| C10EK00 | Type 1 diabetes mellitus with persistent proteinuria |
| C10EC00 | Type 1 diabetes mellitus with polyneuropathy |
| C108012 | Type 1 diabetes mellitus with renal complications |
| C10E700 | Type 1 diabetes mellitus with retinopathy |
| C10E500 | Type 1 diabetes mellitus with ulcer |
| C10EA00 | Type 1 diabetes mellitus without complication |
| 66At011 | Type 1 diabetic dietary review |
| C10F.00 | Type 2 diabetes mellitus |
| C10F700 | Type 2 diabetes mellitus - poor control |
| C10P111 | Type 2 diabetes mellitus in remission |
| C109G12 | Type 2 diabetes mellitus with arthropathy |
| C109E12 | Type 2 diabetes mellitus with diabetic cataract |
| C10FQ00 | Type 2 diabetes mellitus with exudative maculopathy |
| C10F500 | Type 2 diabetes mellitus with gangrene |
| C10FR00 | Type 2 diabetes mellitus with gastroparesis |
| C10FD00 | Type 2 diabetes mellitus with hypoglycaemic coma |
| C10FN00 | Type 2 diabetes mellitus with ketoacidosis |
| C10FP00 | Type 2 diabetes mellitus with ketoacidotic coma |
| C10FA00 | Type 2 diabetes mellitus with mononeuropathy |
| C10F300 | Type 2 diabetes mellitus with multiple complications |
| C10FC00 | Type 2 diabetes mellitus with nephropathy |
| C10F200 | Type 2 diabetes mellitus with neurological complications |
| C10FH00 | Type 2 diabetes mellitus with neuropathic arthropathy |
| C10F100 | Type 2 diabetes mellitus with ophthalmic complications |
| C10FF00 | Type 2 diabetes mellitus with peripheral angiopathy |
| C10FM00 | Type 2 diabetes mellitus with persistent microalbuminuria |
| C10FL00 | Type 2 diabetes mellitus with persistent proteinuria |
| C10FB00 | Type 2 diabetes mellitus with polyneuropathy |
| C109012 | Type 2 diabetes mellitus with renal complications |
| C10F600 | Type 2 diabetes mellitus with retinopathy |
| C10F400 | Type 2 diabetes mellitus with ulcer |
| C10F900 | Type 2 diabetes mellitus without complication |
| 66At111 | Type 2 diabetic dietary review |
| C10E400 | Unstable type 1 diabetes mellitus |
| **SNOMED** | |
| **Code** | **Term** |
| 199223000 | Pre-existing type 1 diabetes mellitus in pregnancy |
| 444074000 | Type 1 diabetes mellitus well controlled |
| 609566000 | Pregnancy and type 1 diabetes mellitus |
| 870528001 | Newly diagnosed type 1 diabetes mellitus |
| 1217068008 | Fulminant type 1 diabetes mellitus |
| 190331003 | Type 2 diabetes mellitus with hyperosmolar coma |
| 199230006 | Pre-existing type 2 diabetes mellitus |
| 237627000 | Pregnancy and type 2 diabetes mellitus |
| 444110003 | Type 2 diabetes mellitus well controlled |
| 1.64971E+14 | Type 2 diabetes mellitus controlled by diet |
| 290002008 | Unstable type I diabetes mellitus |
| 46635009 | Type I diabetes mellitus |
| 190372001 | Type I diabetes mellitus maturity onset |
| 314893005 | Type I diabetes mellitus with arthropathy |
| 420825003 | Type I diabetes mellitus with gangrene |
| 314771006 | Type I diabetes mellitus with hypoglycaemic coma |
| 421468001 | Type I diabetes mellitus with neurological complications |
| 7.1771E+13 | Type I diabetes mellitus with neuropathic arthropathy |
| 3.1211E+13 | Type I diabetes mellitus with peripheral angiopathy |
| 190368000 | Type I diabetes mellitus with ulcer |
| 313435000 | Type I diabetes mellitus without complication |
| 739681000 | Type I diabetes mellitus with ophthalmic complications |
| 422228004 | Type I diabetes mellitus with multiple complications |
| 420789003 | Type I diabetes mellitus with retinopathy |
| 420918009 | Type I diabetes mellitus with mononeuropathy |
| 713705003 | Type I diabetes mellitus with polyneuropathy |
| 421893009 | Type I diabetes mellitus with nephropathy |
| 421920002 | Type I diabetes mellitus with diabetic cataract |
| 420514000 | Type I diabetes mellitus with persistent proteinuria |
| 401110002 | Type I diabetes mellitus with persistent microalbuminuria |
| 420270002 | Type I diabetes mellitus with ketoacidosis |
| 421075007 | Type I diabetes mellitus with ketoacidotic coma |
| 420486006 | Type I diabetes mellitus with exudative maculopathy |
| 7.54101E+14 | Type I diabetic dietary review |
| 713702000 | Type I diabetes mellitus with gastroparesis |
| 703137001 | Type I diabetes mellitus in remission |
| 444073006 | Type I diabetes mellitus uncontrolled |
| 44054006 | Type II diabetes mellitus |
| 314903002 | Type II diabetes mellitus with arthropathy |
| 421631007 | Type II diabetes mellitus with gangrene |
| 719216001 | Type II diabetes mellitus with hypoglycaemic coma |
| 190388001 | Type II diabetes mellitus with multiple complications |
| 314904008 | Type II diabetes mellitus with neuropathic arthropathy |
| 314902007 | Type II diabetes mellitus with peripheral angiopathy |
| 190389009 | Type II diabetes mellitus with ulcer |
| 313436004 | Type II diabetes mellitus without complication |
| 443694000 | Type II diabetes mellitus uncontrolled |
| 237599002 | Insulin treated Type II diabetes mellitus |
| 422099009 | Type II diabetes mellitus with ophthalmic complications |
| 421326000 | Type II diabetes mellitus with neurological complications |
| 422034002 | Type II diabetes mellitus with retinopathy |
| 420436000 | Type II diabetes mellitus with mononeuropathy |
| 713706002 | Type II diabetes mellitus with polyneuropathy |
| 420279001 | Type II diabetes mellitus with nephropathy |
| 420756003 | Type II diabetes mellitus with diabetic cataract |
| 421986006 | Type II diabetes mellitus with persistent proteinuria |
| 420715001 | Type II diabetes mellitus with persistent microalbuminuria |
| 421750000 | Type II diabetes mellitus with ketoacidosis |
| 7.54121E+14 | Type II diabetic dietary review |
| 713703005 | Type II diabetes mellitus with gastroparesis |
| 445353002 | Unstable type II diabetes mellitus |
| 2.4471E+13 | Type II diabetes on insulin |
| 2.4481E+13 | Type II diabetes on diet only |
| **ICD-10** | |
| **Code** | **Term** |
| E100 | Insulin-dependent diabetes mellitus with coma |
| E101 | Insulin-dependent diabetes mellitus with ketoacidosis |
| E102 | Insulin-dependent diabetes mellitus with renal complications |
| E103 | Insulin-dependent diabetes mellitus with ophthalmic comps |
| E104 | Insulin-dependent diabetes mellitus with neurological comps |
| E105 | Insulin-dependent diabetes mellitus with periph circ comps |
| E106 | Insulin-dependent diabetes mellitus with other spec comps |
| E107 | Insulin-dependent diabetes mellitus with multiple comps |
| E108 | Insulin-dependent diabetes mellitus with unspec comps |
| E109 | Insulin-dependent diabetes mellitus without complications |
| E110 | Non-insulin-dependent diabetes mellitus with coma |
| E111 | Non-insulin-dependent diabetes mellitus with ketoacidosis |
| E112 | Non-insulin-dependent diabetes mellitus with renal comps |
| E113 | Non-insulin-dependent diabetes mellitus with ophthalm comps |
| E114 | Non-insulin-dependent diabetes mellitus with neuro comps |
| E115 | Non-insulin-depend diabetes mellitus with periph circ comp |
| E116 | Non-insulin-depend diabetes mellitus with other spec comp |
| E117 | Non-insulin-dependent diabetes mellitus with multiple comps |
| E118 | Non-insulin-dependent diabetes mellitus with unspec comps |
| E119 | Non-insulin-depend diabetes mellitus without complication |
| E130 | Other specified diabetes mellitus with coma |
| E131 | Other specified diabetes mellitus with ketoacidosis |
| E132 | Other specified diabetes mellitus with renal complications |
| E133 | Other specified diabetes mellitus with ophthalmic comps |
| E134 | Other specified diabetes mellitus with neurological comps |
| E135 | Other specified diabetes mellitus with periph circ comps |
| E136 | Other specified diabetes mellitus with other spec comps |
| E137 | Other specified diabetes mellitus with multiple comps |
| E138 | Other specified diabetes mellitus with unspecified comps |
| E139 | Other specified diabetes mellitus without complications |
| E140 | Unspecified diabetes mellitus with coma |
| E141 | Unspecified diabetes mellitus with ketoacidosis |
| E142 | Unspecified diabetes mellitus with renal complications |
| E143 | Unspecified diabetes mellitus with ophthalmic complications |
| E144 | Unspecified diabetes mellitus with neurological comps |
| E145 | Unspecified diabetes mellitus with periph circulatory comps |
| E146 | Unspecified diabetes mellitus with other specified comps |
| E147 | Unspecified diabetes mellitus with multiple complications |
| E148 | Unspecified diabetes mellitus with unspecified complications |
| E149 | Unspecified diabetes mellitus without complications |
| N083 | Glomerular disorders in diabetes mellitus |
| O240 | "Pre-existing diabetes mellitus, insulin-dependent" |
| O241 | "Pre-existing diabetes mellitus, non-insulin-dependent" |
| O242 | "Pre-existing diabetes mellitus, unspecified" |

**Table 5.** Code lists for List of drugs for consider diabetes

| Insulin | Biphasic insulin aspart |
| --- | --- |
|  | Biphasic insulin lispro |
|  | Biphasic isophane insulin |
|  | Insulin aspart |
|  | Insulin degludec |
|  | Insulin detemir |
|  | Insulin glargine |
|  | Insulin glargine/lixisenatide |
|  | Insulin glulisine |
|  | Insulin human |
|  | Insulin Lispro |
|  | Insulin zinc suspension |
|  | Isophane insulin |
|  | Protamine zinc insulin |
|  | Soluble insulin (Neutral insulin) |
| Antidiabetic | Acarbose |
|  | Albiglutide |
|  | Alogliptin |
|  | Alogliptin/metformin |
|  | Canagliflozin |
|  | Canagliflozin/metformin |
|  | Chlorpropamide |
|  | Dapagliflozin |
|  | Dapagliflozin/metformin |
|  | Dulaglutide |
|  | Empagliflozin |
|  | Empagliflozin/linagliptin |
|  | Empagliflozin/metformin |
|  | Ertugliflozin |
|  | Exenatide |
|  | Glibenclamide |
|  | Gliclazide |
|  | Glimepiride |
|  | Glipizide |
|  | Guar gum |
|  | Ins degludec/liraglutide |
|  | Linagliptin |
|  | Linagliptin/metformin |
|  | Liraglutide |
|  | Lixisenatide |
|  | Metformin hydrochloride |
|  | Metformin hydrochloride/pioglitazone |
|  | Metformin hydrochloride/rosiglitazone |
|  | Metformin hydrochloride/sitagliptin |
|  | Metformin hydrochloride/vildagliptin |
|  | Nateglinide |
|  | Pioglitazone hydrochloride  Repaglinide |
|  | Rosiglitazone |
|  | Saxagliptin |
|  | Saxagliptin/dapagliflozin |
|  | Saxagliptin/metformin |
|  | Semaglutide |
|  | Sitagliptin |
|  | Tirzepatide |
|  | Tolbutamide |
|  | Vildagliptin |

**Table 6.** Objective 1: Sensitivity analyses

|  | **IRR (95% CI)** | **p-value** |
| --- | --- | --- |
| **MS diagnosed from 2002 onwards (and their matched controls)** | | |
| Total *n* = 392,146; 36,689 with MS and 355,457 controls | | |
| Unadjusted | 1.10 (1.05 – 1.13) | <0.001* |
| Adjusted | 1.09 (1.03 – 1.11) | <0.001* |
| **Confirmed DM** | | |
| Unadjusted | 1.38 (1.35 – 1.41) | <0.001* |
| Adjusted | 1.22 (1.29 – 1.27) | <0.001* |

**Table 7.** Objective 1: Subgroup analyses

|  | **IRR (95% CI)** | **p-value** |
| --- | --- | --- |
| **Type 1 DM** |  |  |
| Unadjusted | 1.37 (1.12– 2.00) | <0.001* |
| Adjusted | 1.28 (1.01 – 1.87) | <0.001* |
|  |  |  |
| **Type 2 DM** |  |  |
| Unadjusted | 1.03 (0.95 – 1.11) | 0.46 |
| Adjusted | 1.51 (1.07– 1.24) | <0.001* |

**Table 8.** Objective 2: Sensitivity analyses on hospitalisation episodes

|  | **Β (95% CI)** | **p-value** |
| --- | --- | --- |
| **MS diagnosed from 2002 onwards** | | |
| Total *n* = 392,146; 36,689 with MS and 355,457 controls | | |
| Unadjusted | 2.66 (2.25 – 3.15) | <0.001* |
| Adjusted | 1.49 (1.37 – 1.62) | <0.001* |
| **Confirmed DM** | | |
| Unadjusted | 3.39 (2.30– 5.01) | <0.001* |
| Adjusted | 1.28 (1.19– 1.49) | 0.001* |

**Table 9.** Objective 2: Subgroup analyses on hospitalisation episodes

|  | **MS** | | **Controls** | |
| --- | --- | --- | --- | --- |
|  | **IRR (95% CI)** | **p-value** | **IRR (95% CI)** | **p-value** |
| **Type 1 DM** |  |  |  |  |
| Unadjusted | 2.12 (1.79 – 5.11) | 0.08 | 5.19 (3.74 – 7.69) | <0.001* |
| Adjusted | 2.45 (1.96 – 3.99) | <0.001* | 4.36 (2.99 – 6.31) | <0.001* |
|  |  |  |  |  |
| **Type 2 DM** |  |  |  |  |
| Unadjusted | 2.08 (1.79 – 3.11) | <0.001* | 2.79 (2.11 – 3.45) | <0.001* |
| Adjusted | 2.31 (1.88 – 3.38) | <0.001* | 2.01 (1.99 – 3.34) | <0.001* |

**Table 10.** Objective 2: Sensitivity analyses on hospitalisation duration

|  | **Β (95% CI)** | **p-value** |
| --- | --- | --- |
| **MS diagnosed from 2002 onwards** | | |
| Total *n* = 392,146; 36,689 with MS and 355,457 controls | | |
| Unadjusted | 0.96 (0.91 – 1.01) | <0.001* |
| Adjusted | 0.74 (0.61 – 0.87) | <0.001* |
| **Confirmed DM** | | |
| Unadjusted | 1.32 (1.21 – 1.44) | <0.001* |
| Adjusted | 1.24 (0.18 – 0.31) | <0.001* |

**Table 11.** Objective 2: Subgroup analyses on hospitalisation duration

|  | **MS** | | **Controls** | |
| --- | --- | --- | --- | --- |
|  | **Β (95% CI)** | **p-value** | **Β (95% CI)** | **p-value** |
| **Type 1 DM** |  |  |  |  |
| Unadjusted | 1.02 (0.52 – 1.54) | <0.001* | 1.12 (0.91 – 1.37) | <0.001* |
| Adjusted | 1.09 (0.47 – 1.51) | <0.001* | 1.03 (0.65 – 1.21) | <0.001* |
|  |  |  |  |  |
| **Type 2 DM** |  |  |  |  |
| Unadjusted | 1.14 (0.85 – 1.53) | <0.001* | 1.05 (0.91 – 1.11) | <0.001* |
| Adjusted | 1.17 (0.82 – 1.19) | <0.001* | 1.07 (0.81 – 0.99) | <0.001* |

**Table 12**. Objective 3: Subgroup analyses

|  | **MS** | | **Controls** | |
| --- | --- | --- | --- | --- |
|  | **HR (95% CI)** | **p-value** | **HR (95% CI)** | **p-value** |
| **Type 1 DM** |  |  |  |  |
|  | 1.59 (1.08 – 2.34) | 0.01* | 1.57 (1.28– 1.93) | <0.001* |
|  |  |  |  |  |
| **Type 2 DM** |  |  |  |  |
|  | 1.00 (0.89 – 1.14) | 0.89 | 1.09 (0.03 – 1.03) | <0.001* |

**Table 13.** Objective 2: Stratified analyses: Hospitalisation episodes in control group

Stratified by ethnicity

|  | **IRR (95% CI)** | **p-value** |
| --- | --- | --- |
| **White** |  |  |
| Unadjusted | 3.11 (2.38 – 3.46) | <0.001* |
| Adjusted | 2.77 (2.23 – 3.11) | <0.001* |
|  |  |  |
| **Non-White** |  |  |
| Unadjusted | 1.76 (1.45 – 3289) | <0.001* |
| Adjusted | 2.11 (1.98 – 3.01) | <0.001* |

Adjusted for age, gender, IMD, and smoking status

**Table 14.** Objective 2 Stratified analyses: Hospitalisation duration in controls

Stratified by ethnicity

|  | **Β (95% CI)** | **p-value** |
| --- | --- | --- |
| **White** |  |  |
| Unadjusted | 1.04 (1.00 – 1.07) | <0.001* |
| Adjusted | 1.01 (0.98 – 1.23) | <0.001* |
|  |  |  |
| **Non-White** |  |  |
| Unadjusted | 0.92 (0.78 –1.05) | <0.001* |
| Adjusted | 0.88 (0.67 – 0.93) | <0.001* |

Adjusted for age, gender, IMD, and smoking status

**Table 15.** Objective 2: Stratified analyses: Hospitalisation episodes in MS group

Stratified by urbanity

|  | **IRR (95% CI)** | **p-value** |
| --- | --- | --- |
| **Urban** |  |  |
| Unadjusted | 1.34 (1.11– 1.86) | <0.001* |
| Adjusted | 1.59 (1.29 – 2.01) | <0.001* |
|  |  |  |
| **Rural** |  |  |
| Unadjusted | 2.99 (1.90 – 4.11) | <0.001* |
| Adjusted | 2.71 (1.89 – 3.98) | <0.001* |

Adjusted for age, gender, ethnicity, and IMD

**Table 16.** Objective 3 Stratified analyses in controls

Stratified by IMD

|  | **HR (95% CI)** | **p-value** |
| --- | --- | --- |
| **Most deprived** | 0.95 (0.69 – 1.02) | 0.98 |
| **Least deprived** | 1.55 (1.15 – 1.28) | <0.001* |

Adjusted for age, gender, ethnicity, and IMD

**Table 17.** Objective 3 Stratified analyses in controls

Stratified by urbanity

|  | **IRR (95% CI)** | **p-value** |
| --- | --- | --- |
| **Urban** | 1.26 (1.22 – 1.31) | <0.001* |
| **Rural** | 1.48 (1.35 – 1.62) | <0.001* |

Adjusted for age, gender, ethnicity, and IMD

**Figure 1**. Schoenfeld residual plot
